# Examining media’s coverage of COVID-19 vaccines and social media sentiments on vaccine manufacturers’ stock prices

**DOI:** 10.3389/fpubh.2024.1411345

**Published:** 2024-08-13

**Authors:** Shun Yao Bai, Edmund W. J. Lee

**Affiliations:** ^1^College of Computing and Data Science, Nanyang Technological University, Singapore, Singapore; ^2^Department of Media and Communication, City University of Hong Kong, Kowloon, Hong Kong SAR, China

**Keywords:** COVID-19, coronavirus, stock market, social media, sentiment analysis, topic modelling

## Abstract

**Introduction:**

The COVID-19 pandemic caused a widespread public health and financial crisis. The rapid vaccine development generated extensive discussions in both mainstream and social media, sparking optimism in the global financial markets. This study aims to explore the key themes from mainstream media’s coverage of COVID-19 vaccines on Facebook and examine how public interactions and responses on Facebook to mainstream media’s posts are associated with daily stock prices and trade volume of major vaccine manufacturers.

**Methods:**

We obtained mainstream media’s coverage of COVID-19 vaccines and major vaccine manufacturers on Facebook from CrowdTangle, a public insights tool owned and operated by Facebook, as well as the corresponding trade volume and daily closing prices from January 2020 to December 2021. Structural topic modelling was used to analyze social media posts while regression analysis was conducted to determine the impact of Facebook reactions on stock prices and trade volume.

**Results:**

10 diverse topics ranging from vaccine trials and their politicization (note: check that we use American spelling throughout), to stock market discussions were found to evolve over the pandemic. Although Facebook reactions were not consistently associated with vaccine manufacturers’ stock prices, ‘Haha’ and ‘Angry’ reactions showed the strongest association with stock price fluctuations. In comparison, social media reactions had little observable impact on trading volume.

**Discussion:**

Topics generated reflect both actual events during vaccine development as well as its political and economic impact. The topics generated in this study reflect both the actual events surrounding vaccine development and its broader political and economic impact. While we anticipated a stronger correlation, our findings suggest a limited relationship between emotional reactions on Facebook and vaccine manufacturers’ stock prices and trading volume. We also discussed potential technical enhancements for future studies, including the integration of large language models.

## Introduction

The COVID-19 pandemic emerged in December 2019 and quickly spread around the world in the months thereafter, resulting in half a billion cases and millions of confirmed deaths (1). Four years on, 12 vaccines have been developed by various pharmaceutical companies, passed clinical trials and received emergency use authorisation (2). While often compared to the 2009 swine flu and 2002–2004 SARS pandemics, this coronavirus outbreak is unique in various aspects. For instance, advances in medical research and international collaboration allowed for the rapid development of MRNA vaccines that were quickly sent for clinical trials. While vaccine development would normally span a decade or longer, the earliest vaccines by manufacturers like Pfizer were approved by early 2021 (3, 4), slightly over a year since the emergence of the virus.

In the years since earlier pandemics, new media platforms have quickly gained traction, with many Americans switching from traditional print media to digital sources of news. Concurrently, social media giants like Facebook and X (formerly Twitter) gained large userbases which served as live discussion forums as the pandemic spread around the world. On X alone, 2 billion tweets relating to COVID-19 were found (5) as of June 2022. To adapt to this switch, various traditional media outlets regularly post news articles and other content on such user platforms.

Yet, unlike prior pandemics, the high transmissibility and mortality of COVID-19 resulted in a prolonged interest by governments, scientific communities and the general public at large. Accompanying this sustained interest in the pandemic was also an influx of retail investors, presumably driven by media reporting and online discussions on market sentiments. Highly prevalent on X, some have suggested that these discussions were found to be correlated with stock market activity [e.g., (6, 7)]. While there are many studies utilising social media to understand public discussions and amplification of risks [e.g., (8, 9)], few have examined how social media posts correlate with the financial markets, which saw an influx of retail investors rushing in during mandatory lock-downs and the gamification of investing apps. Thus, the aims of this study are to: (a) explore the key themes from mainstream media’s coverage of COVID-19 vaccines on Facebook, and (b) examines how public interactions and responses on Facebook to mainstream media’s posts are associated to daily stock prices and trade volume of major vaccine manufacturers.

## Literature review

Beginning 2020, COVID-19 took centre stage in news reports worldwide. In tracking topic interest in the media, the issue-attention cycle (10) is often discussed. Shih et al. (11) found that this cycle of five stages spanning a pre-problem, alarmed discovery and enthusiasm, realisation of cost of progress, decline of public interest and a post-problem stage was particularly relevant for pandemics. However, in the case of the prolonged COVID-19 outbreak and corresponding media coverage in the United States, traditional issue-attention cycles were not observed by Wirz et al. (12). Similarly in Vietnam, Tran et al. (13) found that topics that captured the most attention on social media changed over time and hypothesised that the issue-attention cycle occurred four times over the two COVID-19 pandemic waves with a small cycle followed by a larger cycle. Hence, it remains unclear if there is a distinct cycle in media coverage with relation to COVID-19.

One of the key applications of tracking topic interest is by the financial sector, where the relationship between media coverage and stock prices and returns has long been a key area of study for many researchers (14, 15). In particular, Tetlock (15) established that media pessimism predicts lower market prices while extreme media pessimism and optimism predict higher trade volumes. While Fang and Peress (16) found that stocks with no media coverage generated higher returns than those with media coverage, subsequent work (17) found that firms with higher visibility were likely to have better corporate governance, sales and productivity growth, implying that the benefits of higher coverage were still ‘inadequately priced’. Hence, while returns were not necessarily positively correlated, relationships between media coverage and both stock price and volume seem to exist.

Early research (18) found that panic generated by media outlets contributed to volatility in sectors perceived to be most affected by the COVID-19 outbreak. Further, the pandemic brought on an influx of retail investors, some of whom were hypothesised to have done so as an alternative avenue to gamble (19). This is consistent with the explanation offered by Zwick (20) that the abstraction of computer-mediated trading has provided investors a means to experience risk as an end in itself. Taken together, these could be plausible reasons that explain an increase in average daily turnover as reported by Chiah and Zhong (19).

To track this topic interest, one widely used tool in social science (21) and communications research (22) is topic modelling with Latent Dirichlet Allocation (LDA) as proposed by Blei et al. (23). It is a form of unsupervised learning to extract key topics discussed from a large corpus of documents with broad applications beyond social science and communication.

In the context of the pandemic and news article modelling however, naïve heuristic-based approaches (24) to determine a mathematically optimal number of topics may not be the most suitable for this context due to the need to retain distinctive topics. Instead, the more common approach in topic modelling, regardless of variation of tool, is to run multiple candidate models with varying numbers of topics to select appropriate models (22).

Structural topic modelling (STM) is one specific extension developed by Roberts et al. (25) that offers researchers the ability to include document-level metadata as possible covariates in the modelling process. Such a result would factor in metadata and variables that could potentially account for topic prevalence and content. Given its relative speed over manual qualitative coding (26) while offering useful tools for analysis of trends over time as documented by researchers like Dehler-Holland et al. (27) and Idler et al. (28) in their analyses of news articles, STM was chosen to analyse the corpus (Table 1).

**Table 1:** Count of articles collected.

Source	Count
ABC News	93
CBS News	285
CNBC	373
CNN	228
Daily Mail	246
Forbes	376
Los Angeles Times	189
NBC News	376
Newsweek	232
POLITICO	132
Reuters	1,310
The New York Times	353
USA TODAY	154
Yahoo Finance	331
Yahoo News	365

Besides traditional media sources like news agencies, the rapid growth of social media platforms such as Facebook and X has allowed for quantitative analysis of public attitudes in the form of topic modelling and sentimental analysis. In fact, most work in this space is interdisciplinary across fields like finance and politics with a focus on Twitter, perhaps in part due to the relative ease of access to its Application Programming Interface (API). A popular and emerging application of this data seeks to predict stock prices given Twitter sentiments. While accuracy varied across methods, trading strategies that incorporate such data have largely outperformed those without corresponding social media data (29). A particularly relevant study by Valle-Cruz et al. (30) found that Twitter accounts of traditional media outlets presented high correlations between Twitter sentiments and stock market behaviour. This sets the stage for the closer examination of social media data.

In the COVID context, these natural language processing approaches to textual data have been successful as well. Zhao et al. (31) was able to extract issues of public concern relating to COVID-19 by examining popular search terms on Chinese social media in the early stage of the pandemic. While various researchers (32, 33) have sought to document COVID-19 topics, much of this work is focused on the early stages of the pandemic, Twitter-only or specific to one country. Thus, there is a unique opportunity to track how the pandemic has evolved over the months based on posts by these mainstream media outlets. Hence, the first research question (RQ) is as follows.

**RQ1:** What were the key topics on COVID-19 vaccines featured by mainstream media?

While much work has been done on Twitter to explore relationships between Twitter sentiments and stock market behaviour, little has been done in relation to Facebook’s data, in part due to a more restrictive API. In recent years, new interactions with posts in the form of reactions ‘Love’, ‘Wow’, ‘Haha’, ‘Sad’, ‘Angry’ and ‘Care’ was launched on the platform, allowing users to an alternative way to interact with posts beyond the standard Facebook ‘Like’. As ‘more deliberate and less automatic communicative behaviours’ than its counterpart ‘Likes’ (34), it was hypothesised that Facebook reactions could be a proxy for sentiments to achieve a similar result in predicting stock market activity.

**RQ2:** What is the relationship between the public’s opinions towards vaccines and the daily stock prices and trade volume of major vaccine manufacturers?

## Methods

### Data collection

The prolonged and global impact of the pandemic is an unparalleled opportunity for us to study how today’s platforms for mass communications could potentially correlate with financial market behaviour.

The major vaccine manufacturers examined are Pfizer ($PFE), Moderna ($MRNA), BioNTech ($BNTX), Johnson & Johnson ($JNJ) and AstraZeneca ($AZN).

As all five manufacturers selected are actively traded on either the New York Stock Exchange [New York Stock (35)] or NASDAQ (36) with three of them being American companies (37–39), the impact is particularly pertinent for US traders. Beyond the US, the impact also extends to the rest of the world as these American stock exchanges are the two largest in the world by both market capitalization and trade volume. For this paper, however, we have restricted the scope the US.

In the US, the Pfizer-BioNTech vaccine was the first to be submitted and obtain the emergency use authorisation, subsequently first to be deployed (40), as well as the first COVID vaccine to submit and obtain full approval from the FDA (41). Moderna followed a similar timeline, mostly in the months after for each stage as the second vaccine to obtain full approval (42). The Johnson & Johnson vaccine did not apply for full approval but was the third to submit for and obtain EUA as well as to be deployed (43). Hence, Pfizer, Moderna, BioNTech and Johnson & Johnson-related keywords were included. During this period (early 2021), the AstraZeneca vaccine, while not authorised for emergency use in the United States (44), also received significant attention for authorisation for use in the United Kingdom, EU and Australia (45). Hence, the inclusion of AstraZeneca.

**Figure 1:**
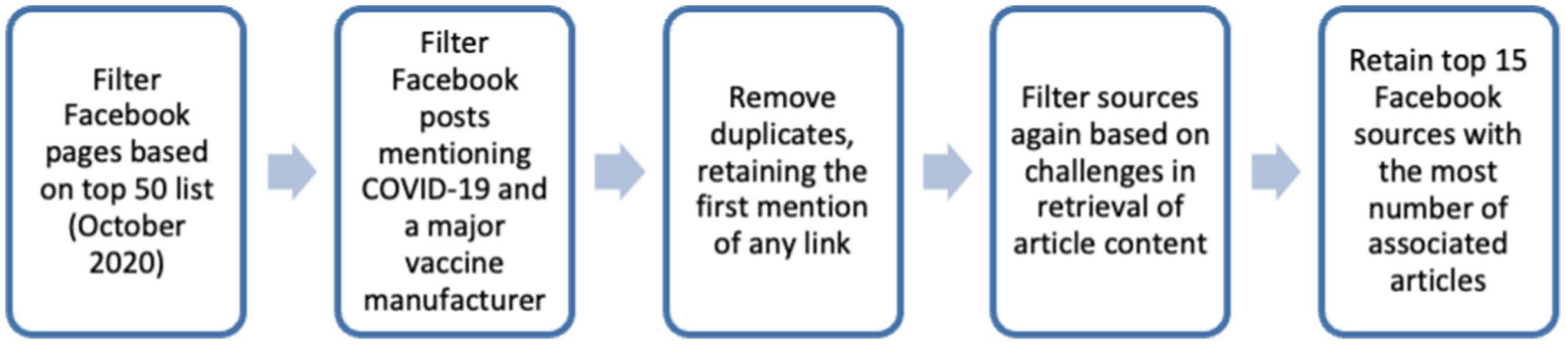
Data collection flowchart.

Data was collected from Facebook, news websites as well the stock market to answer RQ1 and RQ2. For standardisation, a common set of documents and associated posts was used throughout the study. Only articles linked to by Facebook pages of US mainstream media outlets were included. These posts must have included terms relating to coronavirus and a major vaccine manufacturer. The flowchart of corpus selection is as given in Figure 1.

More specifically, news sites were selected from the top 50 media/news websites with the highest readership in the US as listed by PressGazette (46). Facebook posts were filtered from 1 Jan 2020 to 21 Dec 2021 with Facebook’s public insights tool CrowdTangle (47) with the following boolean search parameters (non-case-sensitive).

(ANY: covid, coronavirus, vaccine + ALL: moderna) OR

(ANY: covid, coronavirus, vaccine + ALL: pfizer) OR

(ANY: covid, coronavirus, vaccine + ALL: biontech) OR

(ANY: covid, coronavirus, vaccine + ALL: johnson & johnson) OR

(ANY: covid, coronavirus, vaccine + ALL: AstraZeneca)

Query strings were removed such that a final link can be obtained for each post. Duplicate posts pointing to the same final link would be removed to retain the original link.

As each source structured its content differently, there was a need to verify that the tool in question had successfully collected the article’s information. A naïve rule-based search was implemented by sampling a proportion of articles from each source and filtering for inaccurate content captured.

Owing to various challenges in retrieval of article content, non-existent or inactive Facebook pages, various sites were excluded. The final corpus consisted of 5,043 documents from 15 news outlets.

Stock market information was gathered with the use of Yahoo Finance for the same date range and five vaccine manufacturers.

### Data processing

Following the collection of articles, the corpus was now ready for further analysis. Tokenisation was performed with Quanteda (48) to remove URLs, symbols and separators. A dictionary was implemented to replace proper nouns and its variations to a single word token. To select proper nouns, a recursive n-gram approach was employed. Top 20 n-grams for n between two and five tokens were compiled. Should proper nouns be identified within this list, these were added to the dictionary to be removed.

A regular expression matching alphanumeric characters was then applied to remove non-word tokens before common (R’s stopwords package) and custom stop words (Table 2). Finally, stemming was performed with Quanteda.

**Table 2:** Custom stop words used.

Category	Stop words
Media	reuters, reuterscom, file photo
Vaccines	covid, coronavirus, pandemic, vaccinations, vaccination, vaccines, vaccine, vaccinating, shot, dose, health
Manufacturers	astrazeneca*, pfizer*, moderna*, biontech*, johnson*, covaxin*
Misc.	will, said, people, get, countries, million, use, can, work

*Wildcard matching applied for any trailing text.

### Model building

The corpus was then fed into R’s stm (25) with date as well as source metadata to be used as covariates in order to generate topic models. Beginning with *K* = 4 desired topics, the goal was to identify the model with the most distinct topics according to the independent judgements of two human coders.

For each K desired topic, the top eight tokens of the highest probability, frex, lift and score were given to the human coders. For topics to be considered sufficiently distinct, the coders need to agree upon a similar category for every topic generated for each topic model. Both coders reached a consensus that *K* = 10 generated the greatest number of distinct topics and was hence suitable for the given corpus. The categorisation of the topics and their prevalence can be found in the subsequent Results section.

### Social media and stock market

CrowdTangle information relating to the unique post ID, creation date, sum of all interactions, ‘Likes’, ‘Comments’, ‘Shares’, as well as other reactions of ‘Love’, ‘Wow’, ‘Haha’, ‘Sad’, ‘Angry’ and ‘Care’ were retained. Applying a left join, a simple average was used to aggregate reaction estimates on all Facebook posts for each trading day. Non-trading days were omitted in this analysis.

Following which, regression models were ran on logarithmic-transformed dependent variables trade volume and adjusted close price with scaled CrowdTangle data (R base scale) as independent variables.

## Results

**Figure 2:**
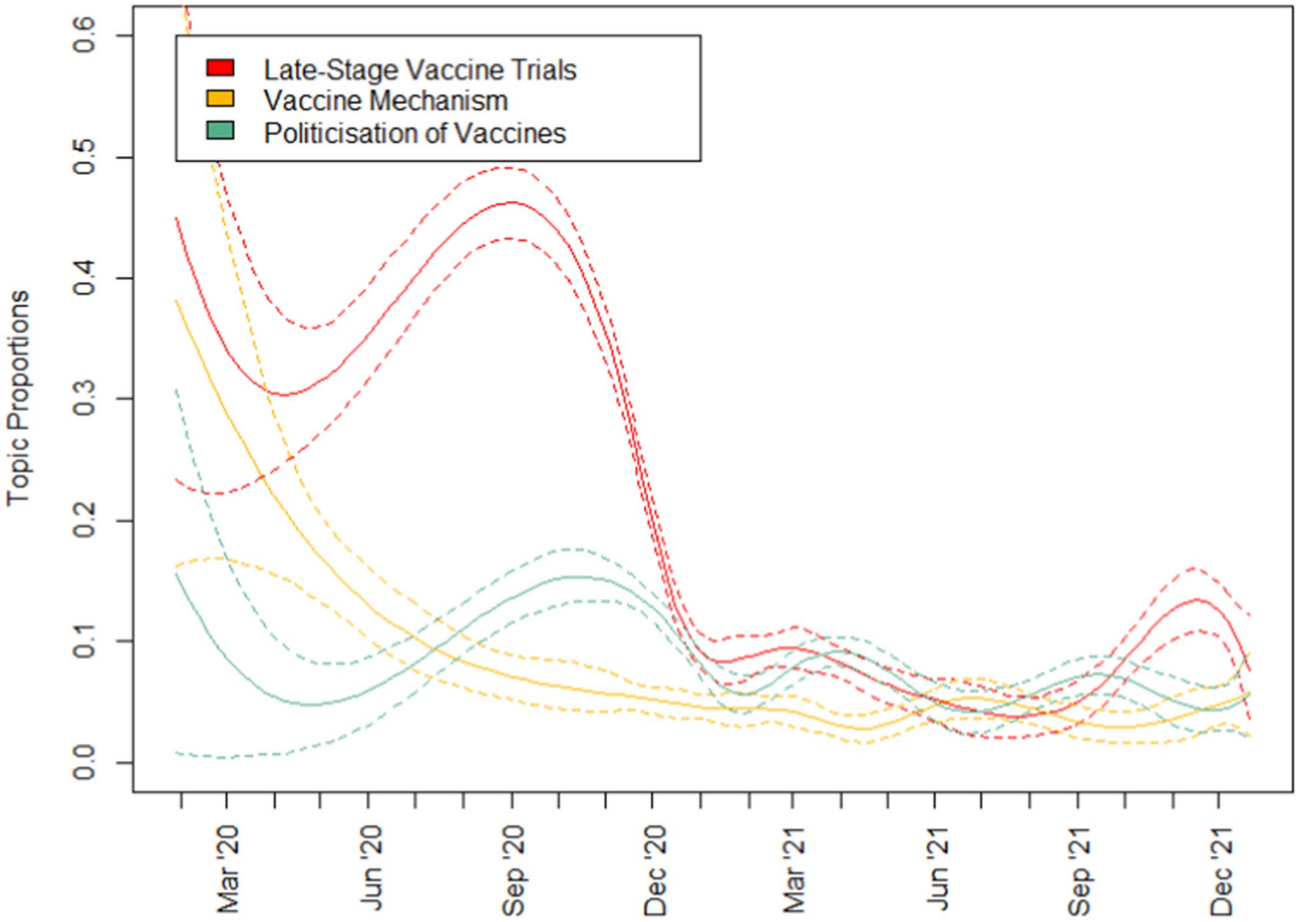
Topic prevalence (1 of 3).

**Figure 3:**
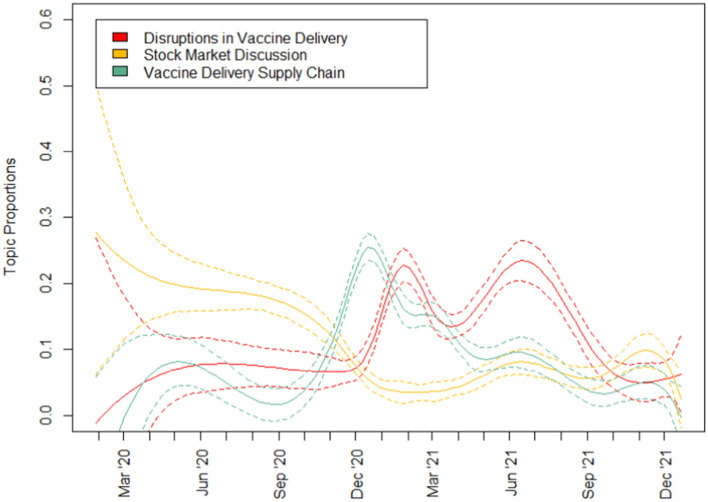
Topic prevalence (2 of 3).

**Figure 4:**
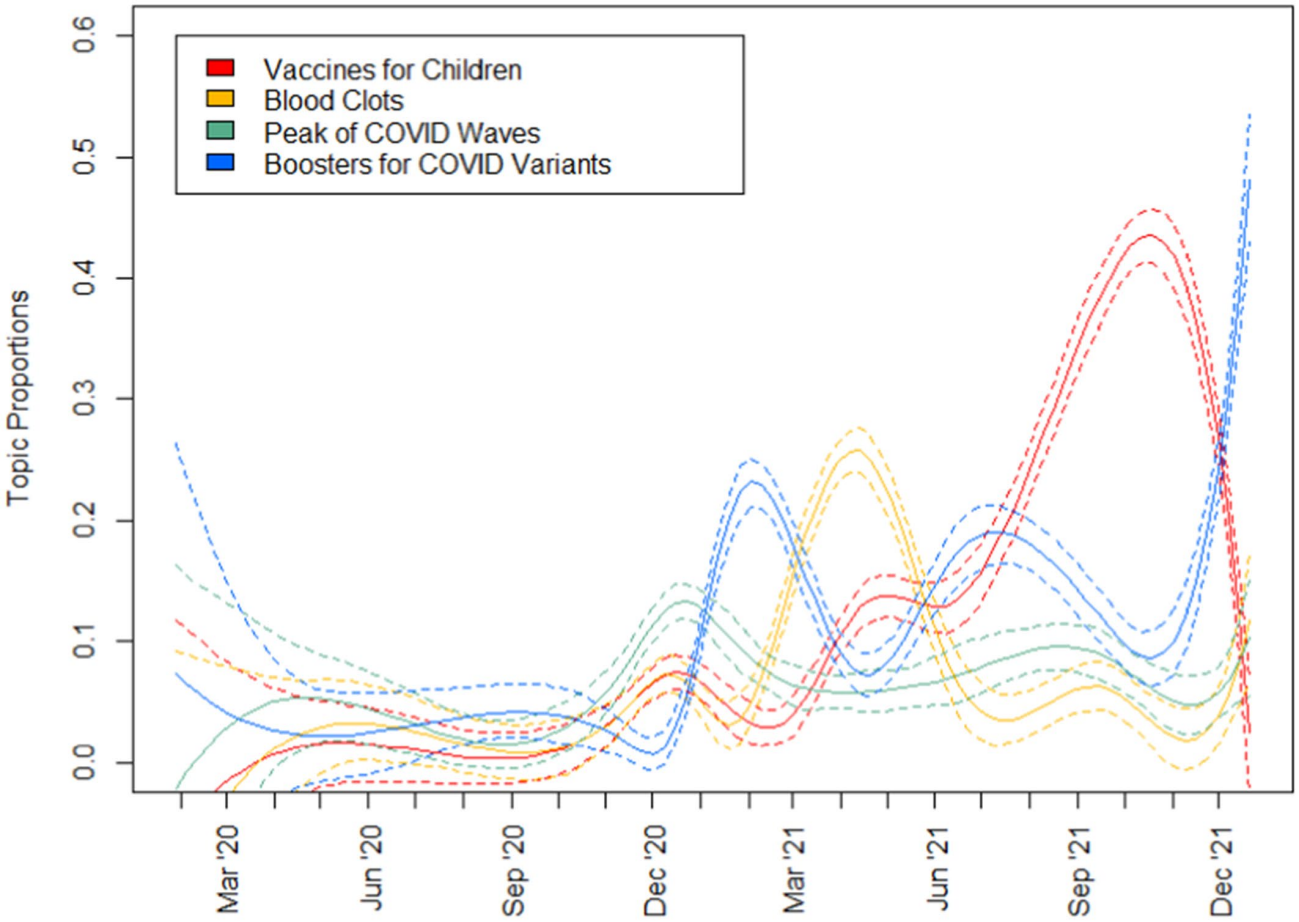
Topic prevalence (3 of 3).

To address RQ1 which aimed to uncover the key topics on COVID-19 as featured in the media, topic modelling was run with results reported in Table 3 and topic prevalence in Figures 2–4. A total of 10 topics were generated encompassing topics relating to (1) late-stage vaccine trials, (2) vaccine mechanism, (3) politicisation of vaccines, (4) disruptions in vaccine delivery, (5) stock market discussions, (6) vaccine delivery supply chain, (7) vaccines for children, (8) blood clots, (9) peak of COVID-19 waves and (10) boosters for COVID-19 variants. Examining the topic prevalence graphs, the results showed that topics varied in prevalence at different stages of the pandemic. For each plot, the 95% confidence interval is given by dotted lines.

**Table 3:** Topics and associated tokens.

	Tokens
**1**	**Late Stage Vaccine Trials**
prob	trial, effect, compani, data, develop, result, approv, particip
frex	late-stag, trial, candid, particip, placebo, interim, volunt, pill
lift	-plus, abbv, acanu, act-acceler, adinarayan, afterhour, age-, aimin
score	trial, candid, late-stag, oxford, placebo, pill, antivir, volunt
**2**	**Vaccine Mechanism**
prob	cell, mrna, virus, protein, spike, immun, develop, technolog
frex	fragment, molecul, helper, tumour, corbett, antigen-, abort, flagship
lift	abortion-deriv, acet, aubrey, aymond, borresen, calquenc, cancer-fight, cation
score	cell, protein, antigen-, fragment, dna, weissman, flagship, adenovirus
**3**	**Politicisation of Vaccines**
prob	dr, fauci, american, public, fda, news, time, trump
frex	fauci, anthoni, trump, polit, black, sharon, tweet, sceptic
lift	a-california, abound, abut, abysm, acquilano, adel, adjut, adweek
score	fauci, trump, fda, biden, dr, hahn, polit, elect
**4**	**Disruptions in Vaccine Delivery**
prob	dose, eu, suppli, govern, india, approv, report, week
frex	export, taiwan, sii, morrison, leyen, delhi, unicef, gavi
lift	-sourc, abu, andrius, ani, anti-wrinkl, baba, baden-wuerttemberg, best-effort
score	eu, taiwan, minist, export, covax, sii, bloc, ministri
**5**	**Stock Market Discussion**
prob	compani, billion, share, stock, market, price, develop, global
frex	zimmer, dow, nasdaq, sec, roe, fiscal, matina, stock
lift	addict, aime, amzn, asx, atherosclerot, bearish, best-sel, bharara
score	billion, patent, price, sale, zimmer, revenu, investor, nasdaq
**6**	**Vaccine Delivery Supply Chain**
prob	dose, distribut, week, fda, emerg, suppli, offici, author
frex	fedex, freezer, ship, dri, temperatur, ice, storag, azar
lift	amerisourc, bayview, behlim, briefcas, cleveng, dept., five-dos, gehm
score	shipment, temperatur, fda, dose, azar, fedex, freezer, perna
**7**	**Vaccines for Children**
prob	booster, fda, children, author, age, shot, data, cdc
frex	adolesc, children, kid, pediatr, teen, panel, booster, cdc
lift	armori, dudley, gaur, guo, honein, −a, −cdc, −health
score	booster, children, fda, cdc, adolesc, myocard, kid, teen
**8**	**Blood Clots**
prob	clot, blood, report, risk, rare, reaction, jab, receiv
frex	clot, platelet, thrombosi, anaphylaxi, cvst, cerebr, thrombocytopenia, causal
lift	anaphylaxi, causal, cerebr, embol, sinus, −arriv, −intern, −iti
score	clot, blood, platelet, ema, allerg, thrombosi, rare, cvst
**9**	**Peak of COVID-19 waves**
prob	hospit, day, test, death, week, vaccin, counti, citi
frex	outdoor, ferrer, fabiano, counti, dine, beach, mayor, keenan
lift	outdoor, aa, aaa, aam, abbey, abdel, aberdeen, abject
score	counti, gov, nhs, student, mayor, blasio, mask, fabiano
**10**	**Boosters for COVID Variants**
prob	variant, studi, protect, effect, infect, virus, data, antibodi
frex	omicron, variant, strain, israel, mutat, isra, delta, neutral
lift	-expert, −studi, −two-dos, abdool, actuari, addedref, additon, adept
score	variant, omicron, booster, delta, studi, antibodi, mutat, neutral

Topics that were more prevalent in 2020 include those relating to vaccine trials, vaccine mechanism, politicisation of vaccination as well as stock market discussions. Following which, topics relating to vaccine delivery disruptions and the supply chain, as well as booster shots and blood clots gained popularity in early 2021. Subsequently, discussions relating to vaccines for children and booster shots peaked in popularity in the latter half of 2022.

For RQ2 aimed to examine the relationship between the public’s opinions towards vaccines and the daily stock prices and trade volume of major vaccine manufacturers, 10 multivariate regression models were run. The major vaccine manufacturers examined were Pfizer (stock ticker $PFE), Moderna ($MRNA), BioNTech ($BNTX), Johnson & Johnson ($JNJ) and AstraZeneca ($AZN).

Table 4 reports coefficients and other statistics when an Ordinary Least Square Regression was run with scaled independent variables likes, comments, shares, reactions and total interactions and logarithmic-transformed dependent variable closing price. Similarly, standardised beta coefficients and other statistics were reported for dependent variable trade volume in Table 5.

**Table 4:** Regression coefficients for closing price.

	Estimate	*p*-value	2.5%	97.5%
**AZN**				
(Intercept)	3.962	0.000	3.955	3.970
Likes	0.004	0.675	−0.016	0.025
Comments	−0.026	0.000	−0.037	−0.014
Shares	0.000	0.902	−0.008	0.007
Love	−0.001	0.881	−0.021	0.018
Wow	−0.010	0.100	−0.023	0.002
Haha	0.032	0.000	0.022	0.041
Sad	0.004	0.426	−0.006	0.015
Angry	0.030	0.000	0.022	0.038
Care	−0.003	0.582	−0.012	0.007
Interactions	−0.006	0.138	−0.014	0.002
**BNTX**				
(Intercept)	4.880	0.000	4.829	4.930
Likes	0.139	0.053	−0.002	0.279
Comments	−0.166	0.000	−0.245	−0.088
Shares	−0.025	0.306	−0.073	0.023
Love	−0.082	0.216	−0.213	0.048
Wow	−0.117	0.006	−0.200	−0.033
Haha	0.285	0.000	0.220	0.350
Sad	0.074	0.044	0.002	0.146
Angry	0.259	0.000	0.202	0.316
Care	0.037	0.267	−0.028	0.102
Interactions	−0.059	0.031	−0.113	−0.005
**JNJ**				
(Intercept)	5.029	0.000	5.022	5.035
Likes	0.021	0.030	0.002	0.040
Comments	−0.013	0.017	−0.023	−0.002
Shares	−0.003	0.373	−0.009	0.004
Love	−0.010	0.273	−0.027	0.008
Wow	−0.007	0.198	−0.019	0.004
Haha	0.024	0.000	0.015	0.033
Sad	0.007	0.142	−0.002	0.017
Angry	0.023	0.000	0.016	0.031
Care	0.003	0.454	−0.005	0.012
Interactions	−0.013	0.000	−0.020	−0.006
**MRNA**				
(Intercept)	4.942	0.000	4.886	4.998
Likes	0.156	0.051	−0.001	0.312
Comments	−0.139	0.002	−0.226	−0.051
Shares	−0.039	0.158	−0.092	0.015
Love	−0.099	0.179	−0.245	0.046
Wow	−0.115	0.016	−0.208	−0.022
Haha	0.269	0.000	0.197	0.341
Sad	0.074	0.071	−0.006	0.154
Angry	0.274	0.000	0.211	0.337
Care	0.064	0.082	−0.008	0.137
Interactions	−0.070	0.023	−0.130	−0.010
**PFE**				
(Intercept)	3.616	0.000	3.603	3.628
Likes	0.039	0.029	0.004	0.074
Comments	−0.042	0.000	−0.061	−0.022
Shares	0.003	0.661	−0.009	0.015
Love	−0.030	0.070	−0.062	0.002
Wow	−0.038	0.000	−0.058	−0.017
Haha	0.069	0.000	0.053	0.085
Sad	0.021	0.019	0.004	0.039
Angry	0.056	0.000	0.041	0.070
Care	0.013	0.103	−0.003	0.030
Interactions	−0.011	0.108	−0.024	0.002

**Table 5:** Regression coefficients for trade volume.

	Estimate	*p*-value	2.5%	97.5%
**AZN**				
(Intercept)	15.700	0.000	15.644	15.756
Likes	0.076	0.341	−0.081	0.233
Comments	0.077	0.084	−0.010	0.165
Shares	−0.039	0.150	−0.093	0.014
Love	−0.048	0.518	−0.194	0.098
Wow	−0.007	0.882	−0.100	0.086
Haha	−0.045	0.225	−0.117	0.028
Sad	0.038	0.358	−0.043	0.118
Angry	−0.039	0.230	−0.102	0.025
Care	0.029	0.432	−0.044	0.102
Interactions	0.009	0.763	−0.051	0.069
**BNTX**				
(Intercept)	14.733	0.000	14.668	14.798
Likes	0.084	0.365	−0.098	0.266
Comments	−0.025	0.623	−0.127	0.076
Shares	−0.006	0.843	−0.068	0.056
Love	0.007	0.931	−0.161	0.176
Wow	−0.130	0.019	−0.238	−0.022
Haha	0.118	0.006	0.034	0.201
Sad	0.070	0.141	−0.023	0.163
Angry	0.125	0.001	0.052	0.199
Care	−0.009	0.835	−0.093	0.076
Interactions	−0.026	0.459	−0.096	0.043
**JNJ**				
(Intercept)	15.746	0.000	15.713	15.778
Likes	0.013	0.777	−0.078	0.104
Comments	0.027	0.307	−0.024	0.077
Shares	0.019	0.231	−0.012	0.050
Love	0.048	0.261	−0.036	0.133
Wow	−0.015	0.579	−0.069	0.039
Haha	−0.020	0.336	−0.062	0.021
Sad	0.040	0.089	−0.006	0.087
Angry	−0.041	0.031	−0.077	−0.004
Care	−0.090	0.000	−0.132	−0.047
Interactions	0.013	0.458	−0.022	0.048
**MRNA**				
(Intercept)	16.353	0.000	16.285	16.420
Likes	0.229	0.018	0.039	0.418
Comments	0.011	0.832	−0.095	0.118
Shares	0.007	0.837	−0.058	0.072
Love	−0.149	0.097	−0.325	0.027
Wow	−0.071	0.216	−0.184	0.042
Haha	−0.070	0.114	−0.157	0.017
Sad	0.020	0.678	−0.077	0.118
Angry	0.014	0.713	−0.062	0.091
Care	0.059	0.190	−0.029	0.147
Interactions	−0.004	0.905	−0.077	0.068
**PFE**				
(Intercept)	17.226	0.000	17.183	17.269
Likes	0.076	0.218	−0.045	0.197
Comments	0.020	0.556	−0.047	0.088
Shares	0.006	0.793	−0.036	0.047
Love	0.016	0.785	−0.097	0.128
Wow	−0.098	0.008	−0.170	−0.026
Haha	0.018	0.520	−0.037	0.074
Sad	0.053	0.096	−0.009	0.115
Angry	0.003	0.920	−0.046	0.051
Care	−0.039	0.172	−0.096	0.017
Interactions	0.027	0.262	−0.020	0.073

When examining coefficients for possible predictors of closing price, Facebook reactions ‘Haha’ (*β* = 0.02–0.29, *p* < 0.001) and ‘Angry’ (*β* = 0.02–0.27, *p* < 0.001) were both positively associated with higher forecast stock prices across all five stocks for 95% confidence intervals. Across all five stocks, these reactions consistently registered the strongest positive relationship with price. In particular, ‘Haha’ reactions had the strongest positive relationship with $BNTX’s price (*β* = 0.29, *p* < 0.001) while ‘Angry’ reactions had the strongest positive relationship with $MNRA’s price (*β* = 0.27, *p* < 0.001). These were significantly larger than other reactions, which were generally <0.10.

In contrast, no single or aggregated metric could be found to predict trade volume across all the same stocks at the same confidence interval. The reaction ‘Haha’ was only correlated for $BNTX (*β* = 0.12, *p* < 0.01) whereas the previously obtained relationship for ‘Angry’ was found for $BNTX (*β* = 0.13, *p* < 0.001). In contrast, the reaction ‘Care’ had a negative relationship with $JNJ’s volume (*β* = −0.09, *p* < 0.001) while ‘Wow’ had a similar negative relationship with $PFE’s volume (*β* = −0.10, *p* < 0.05).

## Discussion

Examining the topics generated (Figures 2–4) to answer RQ1, we see a wide range of topics, with some intertwined with politics and financial markets. Generally, the prevalence of topics seems to be linked to actual events in pandemic and reflective of the media’s coverage and general public’s interest in various aspects of a pandemic. While most of the topics discussed in the media related were exclusive to just vaccines, topics relating to the politicisation of vaccines as well as the stock market discussions were particularly interesting. Unlike other topics, they were not directly linked to the development of vaccines, their safety nor the COVID-19 situation. Instead, these related to completely man-made issues that were not exclusive to the pandemic at all. Discussions relating to these issues were most frequent in 2020, especially in the case of the stock market discussions which largely tapered off in 2021. These could be seen to be issues that generally occurred only once with a significantly smaller or no subsequent recurrence.

While Topics 4 (disruptions in vaccine delivery) and 6 (vaccine delivery supply chain) would be innately related, it is interesting to note that the broader discussions about the supply chain of vaccines peaked in January 2021 only to be followed by two discussions about disruptions in the vaccine delivery. Such a pattern is reminiscent of that discovered by Tran et al. (13) where the cycle could happen multiple times during the same COVID-19 wave.

As vaccines were approved and access to them improved, topics relating to blood clot concerns, boosters and vaccines for children gained popularity in the months after, highlighting a shift in the media’s focus and the public’s interest in these topics. When we examine the relationship between the public’s opinions towards vaccines and the daily stock prices and trade volume of major vaccine manufacturers for RQ2, we find that the general relationship is weak with coefficient estimates of less than 0.25, even with scaled dependent variables.

However, one particular relationship stood out. The diametrically different reactions of ‘Haha’ and ‘Angry’ yielded relatively more pronounced relationships with closing price, suggesting that stronger emotions be linked to higher prices. Owing to the user interface design of reactions on Facebook, users will need to deliberately put in extra effort to interact with posts using these reactions (34). Contrasting this to other reactions of ‘Sad’, ‘Care’, ‘Love’ and ‘Wow’, ‘Haha’ and ‘Angry’ could potentially be the reactions that drive investors and traders to make buy/sell decisions. Another plausible explanation could be a reversal of this explanation in that traders and investors would select these reactions after making such decisions. Alternatively, a bidirectional relationship could be possible as well for a possible direction for further research.

Given the results of closing price, it is noteworthy that a similar outcome was not observed in trade volume; in fact, it seems that there does not exist any common relationship between Facebook metrics and pharmaceutical stocks. As the Facebook metrics are obtained from publicly accessible pages, the underlying assumption that their readers are active traders and investors may not be appropriate, resulting in the results reported. In retrospect, perhaps a more targeted approach could be adopted to answer RQ2. Considerations include a more curated selection of news articles in a social media community more actively engaged in active trading with a more comprehensive API to tap upon for further analyses.

### Limitations

Like all studies, there are several limitations of our study.

First, the catastrophic nature of the pandemic period and its global impact may pose unique challenges in establishing an equal comparison with other periods, all else being equal. However, existing research by Duz Tan and Tas (6), Lazzini et al. (7) and Valle-Cruz et al. (30) found that emotions on social media were partially related to stock market movements, in both pandemic and non-pandemic periods. Thus, we are confident that despite the uniqueness of the pandemic era, we have evidence to show that emotions do relate to market movements in certain contexts.

Second, due to the global nature of social media pages, it would be difficult to draw conclusions based on the viewers of, and those who reacted to, each post. Notwithstanding the inability to obtain specific user demographic data from Facebook, the only way forward for specific user demographic-based analysis would be to engage with each page directly on their user analytics, which would be beyond the scope of this study.

Third, we also note that the data we selected were not representative and thus not generalizable. For instance, the headlines selected to begin with were only a subset of all articles written by the 15 news outlets.

### Future research

For RQ2, we aggregated the public’s opinions towards vaccines by drawing upon the reactions to news outlets’ Facebook posts by members of the general public. To compare 15 news outlets, we were not able to perform a much more granular demographic analysis which could yield deeper and more accurate insight relating to opinions held by the site’s readers. Further research could partner up with social media or Facebook teams directly to obtain the aggregated demographic information of the news outlet’s page viewers, or at a much more granular level, those who interacted with each post. Beyond the Facebook and the news outlets’ own benefit, such a study could also be of interest to sociologists and communications professionals.

Alongside the analysis of Facebook’s reactions, it would also be interesting to consider leveraging large language models (LLMs) as a largely accessible approach for both classification as well as free-form coding tasks in order to analyse comments on such posts. For the former, LLMs could be an easily applicable solution while the latter presents exciting opportunity, having outperformed human coders (49). Such an approach could allow for much greater scale in terms of volume processed with the possibility of generating new insights from user comments.

## Conclusion

This paper sought to examine key topics featured by mainstream media in relation to COVID-19 vaccines as well as to investigate relationships between public perceptions of vaccines and vaccine manufacturers’ stock prices and daily trades.

While distinct topics and trends can be easily identified from the corpus, the relationships between the public’s opinions towards vaccines and the daily stock prices of major vaccine manufacturers were less clear for most reactions. However, the exceptions of reactions ‘Haha’ (*β* = 0.02–0.29, *p* < 0.001) and ‘Angry’ (*β* = 0.02–0.27, *p* < 0.001) were more positively associated with higher forecast stock prices across all five stocks at 95% confidence intervals. While stock prices exhibited stronger relationships, the relationship between the six reactions and trade volume was weaker.

Further expansion to this study could employ more carefully selected datasets with greater considerations to harness the capabilities of natural language processing today, especially with regard to large language models.

Nonetheless, the exploratory analysis in this paper may serve as a primer for future work at the intersection of natural language processing and behavioural finance.

## Data availability statement

The data analysed in this study is subject to the following licenses/restrictions: data from CrowdTangle, a Facebook-owned tool that tracks interactions on public content from Facebook pages and groups, verified profiles, Instagram accounts, and subreddits. It does not include paid ads unless those ads began as organic, non-paid posts that were subsequently “boosted” using Facebook’s advertising tools. It also does not include activity on private accounts, or posts made visible only to specific groups of followers. Requests to access these datasets should be directed to SYB, sbai005@e.ntu.edu.sg.

## Author contributions

SYB: Conceptualization, Data curation, Formal analysis, Investigation, Methodology, Software, Visualization, Writing – original draft, Writing – review & editing. EL: Conceptualization, Formal analysis, Methodology, Supervision, Writing – original draft, Writing – review & editing.
